# Lack of an Association between the SDF-1 rs1801157 Polymorphism and Coronary Heart Disease: A Meta-Analysis

**DOI:** 10.1038/srep11803

**Published:** 2015-07-02

**Authors:** Nan Wu, Xiaowen Zhang, Pengyu Jia, Dalin Jia

**Affiliations:** 1 Department of Cardiology, The First Affiliated Hospital of China Medical University, Liaoning, China; 2 Department of Medical Genetics, China Medical University, Liaoning, China; 3 Department of Clinical Medicine, China Medical University, Liaoning, China

## Abstract

Recent studies have shown that the single-nucleotide polymorphism (SNP) rs1801157 in the stromal cell-derived factor (SDF)-1 gene is associated with susceptibility to coronary heart disease (CHD). However, published studies have shown inconsistent results. Therefore, a meta-analysis was carried out to evaluate the association between rs1801157 and CHD in the literature. A systematic literature search was performed using the PubMed, Web of Science, Cochrane Library, Chinese National Knowledge Infrastructure and Chinese Wan Fang databases. Heterogeneity and publication bias were also evaluated. Seven eligible studies that involved 4656 cases and 2654 controls were finally included in this meta-analysis. Overall, the results showed that the rs1801157 polymorphism was not statistically associated with the risk of CHD under all genetic models but that rs1801157 was associated with decreased susceptibility to myocardial infarction (MI) in subgroup analyses. Moreover, no association was found between rs1801157 and the susceptibility to CHD in either Caucasians or Asians. In conclusion, our meta-analysis demonstrated that the rs1801157 polymorphism is not associated with the susceptibility to CHD but may be associated with a decreased risk of MI. However, further large-scale, case-control studies with rigorous designs should be conducted to confirm these conclusions.

## Introduction

Coronary heart disease (CHD) is becoming one of the major causes of morbidity and mortality worldwide^1^. Multiple factors, such as genetic variants, lifestyle and environmental factors, play critical roles in the occurrence and progression of CHD^2^. A plethora of evidence has demonstrated that atherosclerosis is a major pathologic change in CHD, and inflammatory reactions and immune function disorders are implicated in the development of CHD^3^,^4^. There is evidence that chemokines and critical modulators of inflammatory reactions play key roles in the progression of atherosclerosis and the subsequent onset of CHD^5^,^6^.

Stromal cell-derived factor (SDF)-1 (also called CXCL12) is a small chemokine that usually acts as a chemoattractant to recruit lymphocytes and monocytes^7^ and regulates inflammation, hematopoiesis, embryonic development, tumorigenesis and organ homeostasis^8^,^9^,^10^,^11^. The biologic effects of SDF-1 are mediated by the chemokine receptor CXCR4, a 352-amino-acid rhodopsin-like, transmembrane-specific G protein-coupled receptor^12^. Because lymphocytes and monocytes are involved in the development of atherosclerosis, some researchers have suggested that SDF-1 plays an important role in the pathogenesis of CHD^13^,^14^ and might be a potential biomarker of all-cause mortality^15^.

The gene encoding SDF-1 is located on the human chromosome 10q11.1, which has been previously identified as a susceptibility locus for CHD by genome-wide association studies (GWASs)^16^,^17^. There is evidence that single-nucleotide polymorphism (SNP) loci in the SDF-1 gene, such as rs501120 and rs1746048, are strongly associated with the risk of CHD^16^,^18^. Moreover, a newly found SNP locus in the SDF-1 gene (G801A, rs1801157), which has a G-to-A mutation at position 801 in the 3’-untranslated region, has been shown to upregulate the expression of SDF-1^19^. Most importantly, the rs1801157 polymorphism was shown to be linked to the susceptibility to CHD, but discrepancies exist in the Chinese and Caucasian populations^20^,^21^,^22^. Therefore, in the present study, we performed a meta-analysis to evaluate the association between the rs1801157 polymorphism in the SDF-1 gene and the risk of CHD.

## Methods

### Search strategy

A systematic search was conducted using the PubMed, Web of Science, Cochrane Library, Chinese National Knowledge Infrastructure (CNKI) and Chinese Wan Fang databases until January 1, 2015, to identify all potentially relevant studies. The following search terms were used: (“genetic polymorphism” or “single nucleotide polymorphism” or “SNP” or “gene mutation” or “genetic variants”) and (“coronary atherosclerosis” or “myocardial ischemia” or “acute coronary syndrome” or “coronary disease” or “myocardial infarction” or “ischemic heart disease”) and (“stromal cell-derived factor-1” or “SDF1”or CXCL12” or “chemokine ligand 12” or “rs1801157”). Manual searching was carried out to determine other potentially eligible studies by scanning the references cited in the retrieved articles. The full-text articles were further reviewed to determine whether they could be included in the final analysis strictly based on the eligibility criteria. If two reviewers disagreed, all of the authors critically evaluated the studies to determine whether a certain study should be included or excluded.

### Eligibility criteria

All of the eligible articles had to meet the major inclusion criteria: (i) assessment of the association between the SDF-1 gene polymorphism and CHD; (ii) case-control or cohort studies; and (iii) the data provided concerning allele frequency should be sufficient to calculate genotypic odds ratio (ORs) with the corresponding 95% confidence intervals (95% CIs) in both cases and controls. Studies were excluded when they (i) included duplicated data or (ii) were case reports, letters, review articles or editorial comments. The diagnosis of a CHD case was based on the WHO criteria for CHD, as previously described (stenosis ≥50% of the diameter in at least one major coronary artery based on computer-assisted assessments)^23^,^24^. All of the healthy control subjects were identified according to patient history, serum biochemistry examination and ECG test.

### Data extraction

Data extraction was performed independently by two authors using a standardized data extraction form including the following elements: 1) author’s name, year of publication; 2) patient characteristics of each group; 3) number of participants in the case and control groups; 4) study type; 5) genotyping method; 6) P value of the Hardy–Weinberg equilibrium (HWE) test in the control; and 7) OR and 95% CI for the association with CHD. The study quality was assessed using the Newcastle-Ottawa Scale (NOS), as previously described^25^. Briefly, two authors of this article separately evaluated the study quality based on eight items and assigned a quality score that ranged from 0 to 9 points. Those studies with a score ≥7 points were considered to be of high quality. Any discrepancies were resolved as described above.

### Statistical analysis

First, the genotype frequencies of the rs1801157 polymorphism among the controls of all of the included studies were assessed under HWE using the chi-squared goodness-of-fit test. ORs with their corresponding 95% CIs were used to estimate the strength of the association between the rs1801157 polymorphism and CHD. The between-study heterogeneity across all eligible comparisons was tested using the Cochran’s Q statistic and I-squared (I^2^) metric. Heterogeneity was considered significant with P < 0.10 or I^2^ > 50%. When heterogeneity existed, the random-effects model was performed to calculate the pooled OR of each eligible study; otherwise, the fixed-effect model was used. Generally, we assessed the association between the rs1801157 polymorphism and CHD using five genetic models: allele model (A vs. G), homozygote (co-dominant) model (AA vs. GG), heterozygote (co-dominant) model (AG vs. GG), dominant model (AA/AG vs. GG) and recessive model (AA vs. AG/GG). Subgroup analyses were further performed according to ethnicity (Asian and Caucasian) and primary outcome (CHD and myocardial infarction). Publication bias was analyzed using the Egger’s linear regression test and funnel plots. Publication bias was considered present with P < 0.05. Sensitivity analysis was also performed to evaluate the stability of the meta-analysis. Briefly, a new analysis was performed by omitting one study at a time to test its influence on the overall estimate. All of the statistical analyses were performed using the STATA 11.0 program (STATA Corp., College Station, TX, USA). All of the P values were two-tailed.

## Results

### Characteristics of the included studies

**Figure 1:**
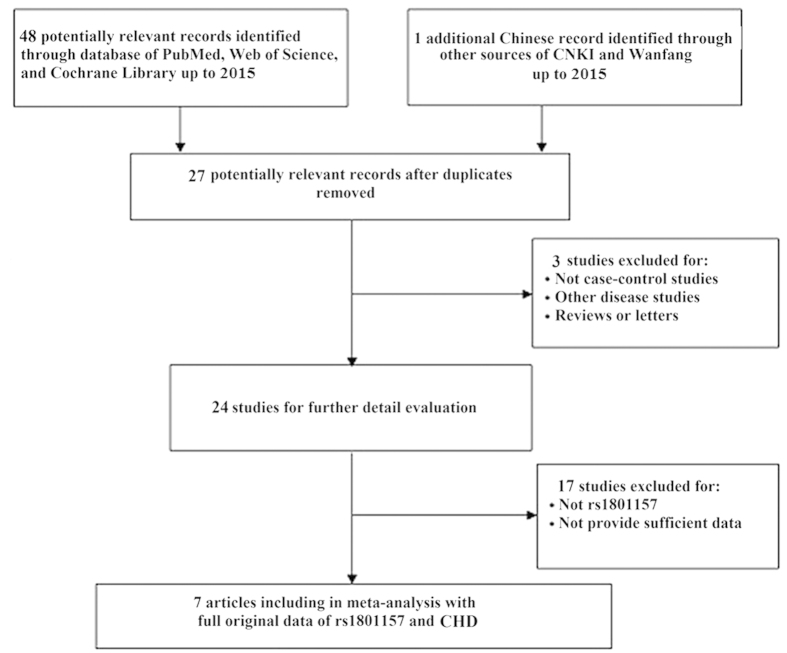
Flow diagram of the study selection process.

As shown in Fig. 1, 49 potentially eligible records were initially identified in the literature search. After different levels of screening, 42 articles were excluded, including 22 articles that were duplicated, 3 articles that did not concern CHD, and 17 articles that did not concern rs1801157. Seven articles were found to be in accordance with the inclusion criteria and were finally included in this meta-analysis^20^,^21^,^22^,^26^,^27^,^28^,^29^.

The characteristics of the included studies are summarized in Table 1. This meta-analysis finally included 7310 subjects (4656 CHD cases and 2654 healthy controls). The genotype distribution of the controls in all of the studies was consistent with HWE. Six of the seven studies used the PCR-RFLP method to detect the rs1801157 polymorphism.

### Quantitative data synthesis

**Figure 2:**
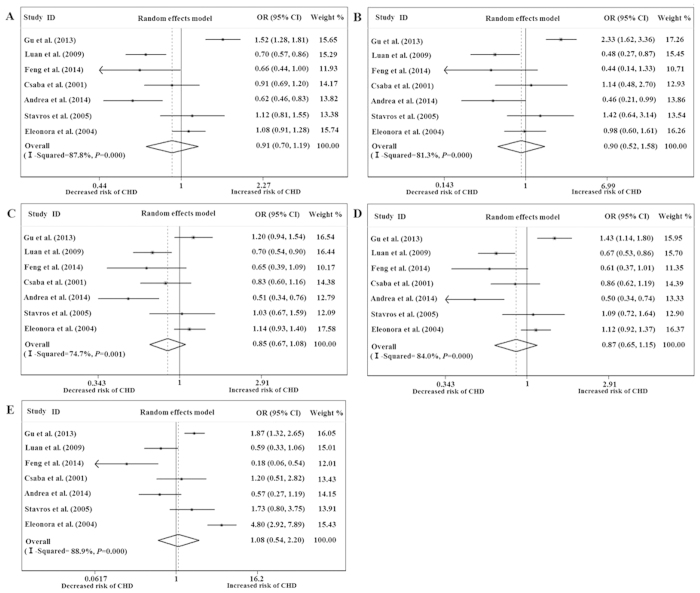
Forests for rs1801157 and coronary heart disease. “**A**” represents allele (A versus G); “**B**” represents homozygote (AA versus GG); “**C**” represents heterozygote (AG versus GG); “**D**” represents dominant (AA/AG versus GG); “E” represents recessive (AA versus AG/GG).

**Figure 3:**
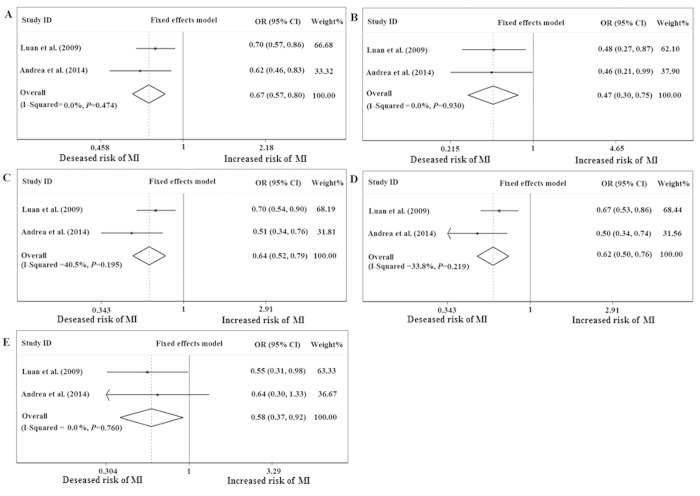
Subgroup analysis of the association between rs1801157 and myocardial infarction. “**A**” represents allele (A versus G); “**B**” represents homozygote (AA versus GG); “**C**” represents heterozygote (AG versus GG); “**D**” represents dominant (AA/AG versus GG); “E” represents recessive (AA versus AG/GG).

No association between the SDF-1 rs1801157 polymorphism and the risk of CHD was found using the five genetic models when all of the data were pooled in the meta-analysis (Fig. 2A–E). We further performed subgroup analysis by primary outcome, and the results showed that there was a significant statistical association between the rs1801157 polymorphism and the risk of myocardial infarction (MI) when using the allele model (A vs. G: OR = 0.674; 95% CI = 0.571–0.797; *P* < 0.001) (Fig. 3A), homozygote model (AA vs. GG: OR = 0.473; 95% CI = 0.297–0.753; *P* = 0.002) (Fig. 3B), heterozygote model (AG vs. GG: OR = 0.640; 95% CI = 0.517–0.792; *P* < 0.001) (Fig. 3C), dominant model (AA/AG vs. GG: OR = 0.618; 95% CI = 0.503–0.759; *P* < 0.001) (Fig. 3D) and recessive model (AA vs. AG/GG: OR = 0.580; 95% CI = 0.368–0.916; *P* = 0.019) (Fig. 3E). However, we found no significant association between the rs1801157 polymorphism and the risk of CHD in either Asians or Caucasians (Table 2).

### Sensitivity analysis

The aim of sensitivity analysis was to evaluate the influence of each study on the pooled ORs and thereby ensure that no single study was completely responsible for the combined results. The results of sensitivity analysis showed that the pooled ORs were not considerably affected by omitting any individual study using the five genetic models, which confirmed that our results were robust (Table 3).

### Publication bias

**Figure 4:**
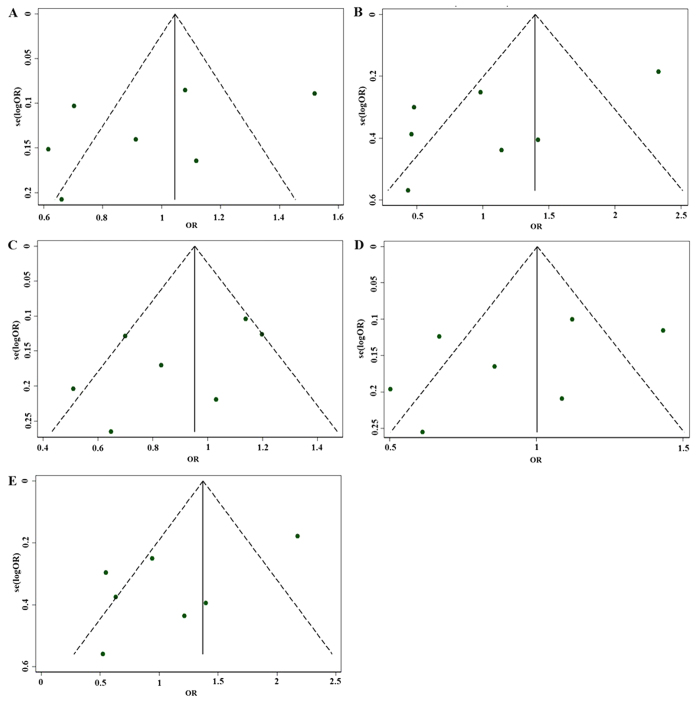
Funnel plots for rs1801157 and coronary heart disease. “**A**” represents allele (A versus G); “**B**” represents homozygote (AA versus GG); “**C**” represents heterozygote (AG versus GG); “**D**” represents dominant (AA/AG versus GG); “**E**” represents recessive (AA versus AG/GG).

Visual inspection of the funnel plot did not reveal any evidence of obvious asymmetry for the five genetic models (Fig. 4A–E). In addition, there was no evidence of publication bias among the studies using all of the genetic models and Egger’s regression test (*P* = 0.243, 95% CI: −14.03181, 4.487158 for the allele model; *P* = 0.086, CI: −11.33623, 1.042932 for the homozygote model; *P* = 0.183, 95% CI: −8.048598, 2.003117 for the heterozygote model; *P* = 0.234, 95% CI: −10.6939, 3.317689 for the dominant model; *P* = 0.093, 95% CI: −10.0156, 1.077358 for the recessive model), which suggested that no publication bias existed.

## Discussion

SDF-1 was demonstrated to be a chemokine that exerts protective effects on the pathogenesis of CHD^30^,^31^. Zernecke *et al.* reported that SDF-1 recruited circulating neutrophils to atherosclerotic lesions, whereas the depletion of neutrophils reduced plaque formation and prevented its exacerbation^29^. Damas *et al.* found that reduced SDF-1 plasma levels were associated with unstable coronary artery disease in a clinical study and suggested that plasma SDF-1 might mediate anti-inflammatory and matrix-stabilizing effects in unstable angina^31^. In addition, several studies have confirmed that SDF-1 conferred myocardial protection in myocardial infarction by modulating ischemia-reperfusion injury^32^,^33^,^34^.

The SDF-1 rs1801157G/A polymorphism resides in a hot SNP locus that has been reported in diverse research fields and is associated with cancer, dermatosis and infectious disease^35^,^36^,^37^,^38^. Several GWASs have confirmed that the rs501120 and rs1746048 polymorphism loci in the SDF-1 gene are associated with the susceptibility to CHD^16^,^17^, but rs1801157 was not reported in any of the above studies. The cause may be that GWASs cannot identify all of the SNPs involved in a single action, although they could offer a large amount of information on SNPs^20^. Furthermore, because the rs1801157 polymorphism was demonstrated to upregulate the expression of SDF-1^19^, some researchers have suggested that rs1801157 is associated with a decreased risk of CHD^20^,^26^,^27^. However, some controversies remain in the literature concerning the relationship between the rs1801157 polymorphism and risk of CHD. Szalai *et al.*, Apostolakis *et al.*, and Simeoni *et al.* suggested that there was no correction between rs1801157 and the risk of CHD^22^,^28^,^29^. In contrast, four other studies reported that rs1801157 was associated with susceptibility to CHD^20^,^21^,^26^,^27^. Of these four studies, Gu *et al.* suggested that rs1801157 was associated with increased susceptibility to coronary artery disease^21^, but the three other studies reported that rs1801157 was associated with a decreased risk of CHD^20^,^26^,^27^. Based on these contradictory results, meta-analysis seemed to be a good approach to combine the results of various studies on the same topic and to further estimate and explain their diversity^39^.

To our knowledge, our study was the first report to pool published case-control studies to estimate the association between the rs1801157 polymorphism and susceptibility to CHD. The result of the meta-analysis showed that the rs1801157 polymorphism was not associated with the risk of CHD, but the result also yielded significant heterogeneity across studies. To explore the source of heterogeneity, we further performed subgroup and sensitivity analyses. The results of subgroup analysis not only suggested that rs1801157 was significantly associated with a decreased risk of MI but also significantly diminished the heterogeneity across studies, which indicated that the difference in CHD subtype was a source of heterogeneity. However, sensitivity analysis did not identify any sources of heterogeneity.

Several limitations existed in our meta-analysis. First, only seven published studies involving a total of 7310 subjects were included in the final meta-analysis. Similarly, only two studies with a small sample size were involved in our analysis of the association between this polymorphism and susceptibility to MI. The sample size remained relatively small and may not exactly estimate the correlation between the rs1801157 polymorphism and susceptibility to CHD or MI. Therefore, more studies with a larger sample size should be included to enhance the reliability and stability of the meta-analysis.

Second, strong heterogeneity exists in the meta-analysis of the association between the rs1801157 polymorphism and the risk of CHD. However, we did not perform meta-regression analysis to explore the source of heterogeneity because meta-regression analysis is not suitable for assessing heterogeneity with a sample size <10^40^.

Finally, although MI was considered a subtype of CHD for subgroup analysis, more subtypes of CHD, such as stable angina and acute coronary syndrome, should be further analyzed. However, we could not analyze the difference among more subtypes of CHD because of the lack of sufficient statistical data in the literature.

In conclusion, our meta-analysis suggested that the SDF-1 rs1801157 polymorphism is not associated with the susceptibility to CHD but may be associated with a decreased risk of MI. Further large-scale, case-control studies with rigorous designs should be conducted to confirm the above conclusions. Despite some limitations, this meta-analysis still provides new insights into the role of the SDF-1 gene in the occurrence and progression of CHD.

## Additional Information

**How to cite this article**: Wu, N. *et al.* Lack of an Association between the SDF-1 rs1801157 Polymorphism and Coronary Heart Disease: A Meta-Analysis. *Sci. Rep.* **5**, 11803; doi: 10.1038/srep11803 (2015).

## Supplementary Material

Supplementary Information

## Figures and Tables

**Table 1:** Main characteristics of studies included in the meta-analysis.

Studies	Country	Ethnicity	Number	Age, year	Male%	Hypertesion%	Smoking%	Diabetes%	hyperlipidemia%	Study type	Primary	Genotype	NOS	HWE test
			(case/control)	(case/control)	(case/control)	(case/control)	(case/control)	(case/control)	(case/control)		outcome	method	score	(control)
Gu *et al*.(2013)	China	Asian	592/625	56.6/55.7	60.0/56.0	46.8/15.2	42.4/14.2	33.6/10.2	NA	Case-control study	CHD	PCR-RFLP	9	Yes
Luan *et al*.(2009)	China	Asian	560/532	55.4/55.1	73.4/73.3	53.8/47.2	56.8/36.5	NA	NA	Case-control study	MI	PCR-RFLP	9	Yes
Feng *et al*.(2014)	China	Asian	84/253	55/45	78.6/60.1	61.9/10.3	NA	NA	NA	Case-control study	CHD	MassARRAY system	9	Yes
Csaba *et al*.(2001)	Hungary	Caucasian	318/320	57.6/58.9	76.1/75	53.1/0.0	66/NA	NA	NA	Case-control study	CHD	PCR-RFLP	9	Yes
Andrea *et al*.(2014)	Italy	Caucasian	200/230	57.3/52	90.5/42	44.9/25	69.5/28	15.0/3.0	74.0/24.0	Case-control study	MI	PCR-RFLP	9	Yes
Stavros *et al*.(2005)	Greece	Caucasian	208/164	63.7/63.2	77.6/76.4	67.1/54.5	57.6/42.4	34.3/16.4	75.2/59.4	Case-control study	CHD	PCR-RFLP	9	Yes
Eleonora *et al*.(2004)	Germany	Caucasian	2694/530	63.8/56.9	73.9/51.3	61.9/42.1	67.7/46.4	22.5/7.5	68.4/36.6	Case-control study	CHD	PCR-RFLP	9	Yes

HWE: Hardy–Weinberg equilibrium; NA: data is not available.

**Table 2:** Main results of the meta-analysis of the pooled OR.

Variable	Cases/controls (n)	OR^b^ (95% CI) Ph value				
		Allele(A vs.G)	Homozygote(AA vs.GG)	Heterzygote(AG vs.GG)	Dominant(AA/AG vs.GG)	Recessive(AA vs.AG/GG)
All subjects	4656/2654	0.914(0.704–1.186)0.499	0.903(0.516–1.581)0.722	0.854(0.675–1.081)0.189	0.865(0.654–1.145)0.312	0.982(0.609–1.583)0.939
Primary outcome						
CHD	3896/1892	1.056(0.826–1.351)0.663	1.223(0.716–2.090)0.461	1.053(0.924–1.199)^a^0.441	1.036(0.814–1.320)0.772	1.244(0.766–2.021)0.377
MI	760/762	0.674(0.571–0.797)^a^0.000	0.473(0.297–0.753)^a^0.002	0.640(0.517–0.792)^a^0.000	0.618(0.503–0.759)^a^0.000	0.580(0.368–0.916)^a^0.019
Ethnicity						
Asian	1236/1410	0.903(0.500–1.630)0.735	0.830(0.237–2.905)0.771	0.839(0.553–1.271)0.407	0.855(0.477–1.534)0.600	0.905(0.304–2.692)0.858
Caucasian	3420/1244	0.916(0.712–1.179)0.497	0.922(0.661–1.285)^a^0.632	1.140(0.929–1.398)0.378	0.866(0.615–1.219)0.408	0.971(0.698–1.351)^a^0.862

Ph, P value for Cochran’s Q test for between-study heterogeneity in each genetic comparison model. a: A fixed effects model was used when the P value for Cochran’s Q test for heterogeneity >0.1. Otherwise, a random effects model was used. b: Crude OR.

**Table 3:** Sensitivity analysis.

Study omitted	Cases/controls (n)	Crude OR 95%CI				
		Allele (A vs.G)	Homozygote (AA vs.GG)	Heterzygote (AG vs.GG)	Dominant (AA/AG vs.GG)	Recessive (AA vs.AG/GG)
Gu *et al*. (2013)	592/625	0.86(0.68,1.04)	0.83(0.52,1.14)	0.84(0.62,1.05)	0.83(0.61,1.05)	0.87(0.59,1.15)
Luan *et al*. (2009)	560/532	1.01(0.72,1.29)	1.18(0.47,1.89)	0.94(0.73,1.16)	0.97(0.70,1.25)	1.20(0.58,1.82)
Feng *et al*. (2014)	84/253	1.00(0.71,1.28)	1.15(0.44,1.87)	0.92(0.71,1.14)	0.96(0.68,1.24)	1.17(0.54,1.80)
Csaba *et al*. (2001)	318/320	0.96(0.66,1.27)	1.06(0.30,1.81)	0.90(0.67,1.14)	0.93(0.63,1.23)	1.08(0.41,1.74)
Andrea *et al*. (2014)	200/230	1.01(0.73,1.29)	1.17(0.45,1.89)	0.96(0.76,1.15)	0.99(0.72,1.25)	1.17(0.53,1.82)
Stavros *et al*. (2005)	208/164	0.93(0.63,1.23)	1.01(0.24,1.78)	0.88(0.65,1.10)	0.89(0.60,1.19)	1.05(0.37,1.72)
Eleonora *et al*. (2004)	2694/530	0.93(0.59,1.27)	1.08(0.27,1.89)	0.84(0.62,1.07)	0.88(0.55,1.20)	1.12(0.42,1.82)

Abbreviation: OR, odds ratio; 95%CI, 95% confidence interval.
