# Supplementary material for: Lack of an Association between the SDF-1 rs1801157 Polymorphism and Coronary Heart Disease: A Meta-Analysis
Source: Sci Rep. 2015 Jul 2;5:11803. doi: 10.1038/srep11803 (PMC4488865; doi:10.1038/srep11803)
Supplement: Supplementary Information [file srep11803-s1.pdf]

Lack of an Association between the SDF-1 rs1801157 Polymorphism and Coronary Heart Disease: A Meta-Analysis

Nan Wu[1#], Xiaowen Zhang[2#], Pengyu Jia[3], Dalin Jia[1]*

Table S1.Details for excluded studies

Title	First author	Journal	database	year	excluded reason
Stromal cell-derived factor 1 as a biomarker of heart failure and mortality risk.	Subramanian S	Arterioscler Thromb Vasc Biol.	Pubmed	2014	not rs1801157
Analysis of common and coding variants with cardiovascular disease in the Diabetes Heart Study.	Adams JN	Cardiovasc Diabetol	Pubmed	2014	not rs1801157
Genetic variants associated with myocardial infarction and the risk factors in Chinese population.	Wang Y	PLoS One	Pubmed	2014	not rs1801157
A novel polymorphism (901G > a) of C5L2 gene is associated with coronary artery disease in Chinese Han and Uyghur population.	Zheng YY	Lipids Health Dis	Pubmed	2013	not rs1801157
Relationship between chemokine (C-X-C motif) ligand 12 gene variant (rs1746048) and coronary heart disease: case-control study and meta-analy	Huang Y	Gene	Pubmed	2013	not rs1801157
RNAi-based functional profiling of loci from blood lipid genome-wide association studies identifies genes with cholesterol-regulatory function.	Blattmann P	PLoS Genet	Pubmed	2013	other disease
Lack of association between the CXCL12 rs501120 polymorphism and cardiovascular disease in Spanish patients with rheumatoid arthritis.	López-Mejías R	Hum Immunol	Pubmed	2012	not rs1801157
Large scale association analysis identifies three susceptibility loci for coronary artery disease.	Saade S	PLoS One	Pubmed	2011	not rs1801157
Association of genetic variants and incident coronary heart disease in multiethnic cohorts: the PAGE study.	Franceschini N	Circ Cardiovasc Genet	Pubmed	2011	not rs1801157
Comparative analysis of genome-wide association studies signals for lipids, diabetes, and coronary heart disease: Cardiovascular Biomarker Genetics	Angelakopoulou A	Eur Heart J	Pubmed	2012	not rs1801157
The novel atherosclerosis locus at 10q11 regulates plasma CXCL12 levels.	Mehta NN	Eur Heart J	Pubmed	2011	not rs1801157
Subclinical atherosclerosis and genetic risk markers in healthy offspring of patients with premature myocardial infarction.	Barra S	Minerva Cardioangiol	Pubmed	2011	not rs1801157
Effect of CC chemokine receptor 2 CCR2 blockade on serum C-reactive protein in individuals at atherosclerotic risk and with a single nucleotide polymorphism of the monocyte chemoattractant protein-1 promoter region.	Gilbert J	Am J Cardiol	Pubmed	2011	not rs1801157
Genetic risk score and risk of myocardial infarction in Hispanics.	Qi L	Circulation	Pubmed	2011	not rs1801157
Coronary artery disease-related genetic variant on chromosome 10q11 is associated with carotid intima-media thickness and atherosclerosis.	Kiechl S	Arterioscler Thromb Vasc Biol	Pubmed	2010	not rs1801157
Genome-wide association of early-onset myocardial infarction with single nucleotide polymorphisms and copy number variants.	Myocardial Infarction Genetics Conso	Nat Genet	Pubmed	2009	not rs1801157
Stromal cell-derived factor 1 as a biomarker of heart failure and mortality risk.	Subramanian S	Arterioscler Thromb Vasc Biol.	Web of sci	2014	duplication
Analysis of common and coding variants with cardiovascular disease in the Diabetes Heart Study.	Adams JN	Cardiovasc Diabetol	Web of sci	2014	duplication
Genetic variants associated with myocardial infarction and the risk factors in Chinese population.	Wang Y	PLoS One	Web of sci	2014	duplication
Genetic Variants on Chromosome 10q11.21 are Associated with Ischemic Stroke in the Northern Chinese Han Population	Zhu Ruixia	JOURNAL OF MOLECULAR NEUROS	Web of sci	2013	other disease
Particle swarm optimization algorithm for analyzing SNP-SNP interaction of renin-angiotensin system genes against hypertension	Wu Shyh-Jong	MOLECULAR BIOLOGY REPORTS	Web of sci	2013	other disease
Relationship between chemokine (C-X-C motif) ligand 12 gene variant (rs1746048) and coronary heart disease: case-control study and meta-analy	Huang Y	Gene	Web of sci	2013	duplication
RNAi-based functional profiling of loci from blood lipid genome-wide association studies identifies genes with cholesterol-regulatory function.	Blattmann P	PLoS Genet	Web of sci	2013	duplication
Lack of association between the CXCL12 rs501120 polymorphism and cardiovascular disease in Spanish patients with rheumatoid arthritis.	López-Mejías R	Hum Immunol	Web of sci	2012	duplication
Comparative analysis of genome-wide association studies signals for lipids, diabetes, and coronary heart disease: Cardiovascular Biomarker Genetics	Angelakopoulou A	Eur Heart J	Web of sci	2012	duplication
Large scale association analysis identifies three susceptibility loci for coronary artery disease.	Saade S	PLoS One	Web of sci	2011	duplication
Association of genetic variants and incident coronary heart disease in multiethnic cohorts: the PAGE study.	Franceschini N	Circ Cardiovasc Genet	Web of sci	2011	duplication
Association of genetic variants and incident coronary heart disease in multiethnic cohorts: the PAGE study.	Franceschini N	Circ Cardiovasc Genet	Web of sci	2011	duplication
The novel atherosclerosis locus at 10q11 regulates plasma CXCL12 levels.	Mehta NN	Eur Heart J	Web of sci	2011	duplication

Subclinical atherosclerosis and genetic risk markers in healthy offspring of patients with premature myocardial infarction.	Barra S	Minerva Cardioangiol	Web of sci	2011	duplication
Effect of CC chemokine receptor 2 CCR2 blockade on serum C-reactive protein in individuals at atherosclerotic risk and with a single nucleotide polymorphism of the monocyte chemoattractant protein-1 promoter region.	Gilbert J	Am J Cardiol	Web of sci	2011	duplication
Common genetic polymorphisms in Moyamoya and atherosclerotic disease in Europeans	Roder C	CHILDS NERVOUS SYSTEM	Web of sci	2011	not rs1801157
Genetic risk score and risk of myocardial infarction in Hispanics.	Qi L	Circulation	Web of sci	2011	duplication
Coronary artery disease-related genetic variant on chromosome 10q11 is associated with carotid intima-media thickness and atherosclerosis.	Kiechl S	Arterioscler Thromb Vasc Biol	Web of sci	2010	duplication
Genome-wide association of early-onset myocardial infarction with single nucleotide polymorphisms and copy number variants.	Myocardial Infarction Genetics Conso	Nat Genet	Web of sci	2009	duplication
Genome-wide association of early-onset myocardial infarction with single nucleotide polymorphisms and copy number variants.	Myocardial Infarction Genetics Conso	Nat Genet	Web of sci	2009	duplication
多个候选基因单核苷酸多态性与冠心病的关联分析	黄毅		CNKI	2013	not rs1801157
Stromal cell-derived factor-1-3 ' A polymorphism is associated with decreased risk of myocardial infarction and early endothelial disturbance	Borghini, A	JOURNAL OF CARDIOVASCULAR MI	Web of sci	2014	duplication
A Single Nucleotide Polymorphism in the Stromal Cell-Derived Factor 1 Gene Is Associated with Coronary Heart Disease in Chinese Patients	Feng, L	INTERNATIONAL JOURNAL OF MOL	Web of sci	2014	duplication
Polymorphism of stromal cell-derived factor-1 selectively upregulates gene expression and is associated with increased susceptibility to coronary artery disease	Gu, XL	BIOCHEMICAL AND BIOPHYSICAL I	Web of sci	2014	duplication
Association of the SDF1-3'A polymorphism with susceptibility to myocardial infarction in Chinese Han population	Luan, B	MOLECULAR BIOLOGY REPORTS	Web of sci	2010	duplication
Involvement of polymorphisms in the chemokine system in the susceptibility for coronary artery disease (CAD). Coincidence of elevated Lp(a) and MCP-1-2518 G/G genotype in CAD patients	Szalai, C	ATHEROSCLEROSIS	Web of sci	2001	duplication
